# Implementing a Co‐Produced Women's Health Programme in an Integrated Care System

**DOI:** 10.1002/puh2.70223

**Published:** 2026-04-17

**Authors:** Susan Conquer, Lizzie Mapplebeck, Annetta Bradshaw, Lisa Nobes, Camille Cronin

**Affiliations:** ^1^ University of Suffolk & Suffolk and North East Essex ICB Suffolk UK; ^2^ Suffolk and North East Essex ICB Suffolk UK; ^3^ Healthwatch Suffolk Suffolk UK; ^4^ School of Health and Social Care University of Essex Colchester Essex UK

**Keywords:** co‐design, community engagement, co‐production, public patient involvement and engagement, service improvement, women's health

## Abstract

**Introduction:**

Co‐production is a key principle of the Women's Health Strategy for England, yet there are limited published examples of its application at system level. This article describes the development of a co‐produced women's health programme within an integrated care system in England.

**Methods:**

A service improvement approach was used, involving a multidisciplinary advisory group of over 160 members, including people with lived experience. The group co‐designed methods to identify local priorities and support programme development.

**Results:**

A survey of 1238 respondents identified gaps in access to and integration of women's health services. These findings informed the design and implementation of five prioritised women's health service models, each with defined objectives and built‐in evaluation to support quality and sustainability.

**Discussion:**

This study demonstrates how community co‐production can inform the design of locally responsive women's health services. Integrating lived experience with professional expertise supported equitable service development, though challenges included limited long‐term funding and sustaining broad engagement.

**Conclusion:**

System‐level co‐production can support the development of women's health programmes aligned with national strategy. This model illustrates a co‐production approach that may inform the development of women's health services in other local settings.

**Patient and Public Contribution:**

People with lived experience of women's health services were involved throughout, including identifying priorities, co‐designing solutions and shaping programme development.

## Graphical abstract

A co‐produced women's health programme is implemented within an integrated care system using a structured, six‐phase service improvement approach. Engagement with over 1200 participants identifies barriers, priorities, and service gaps. Findings inform the co‐design of five targeted service models. Integrating lived experience with professional expertise supports equitable, accessible, and sustainable women's health services aligned with national strategy.

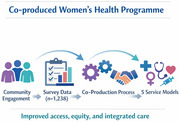

## 1 Introduction

The call for a more person‐centred and inclusive approach for women in health service provision has gained prominence. This followed the largest and most momentous parliamentary consultation in England in 2022 which looked at what women need in terms of health and social care. In the call for evidence, there are many reported cases where ‘*women* *’s voices have not been listened to—indeed the responses to our call for evidence found 84% of respondents felt that this was the case*’ [1]. Many activists for women's rights, particularly around women's health, saw this as a significant milestone [2] and saw the publication of the first Women's Health Strategy for England [1].

Women frequently report barriers to timely, appropriate care and describe not feeling listened to [1]. Given the increasing concerns around women's health and raised profile discussions around HRT use and menopause [3], there is a need to review and evaluate the current care provision in local health services [4].

Women's sexual and reproductive health needs are complex and vary across the life course [2]. They are met by a range of healthcare providers, professionals across a range of venues. Provision is not well integrated, with inequalities in access. The World Health Organization [5] has advocated a life‐course approach to health and wellbeing (including sexual health and wellbeing) by ensuring that women have access to appropriate health information and services to promote healthy ageing and a high quality of life.

Working in partnership with people and communities is a statutory duty within the Health and Care Act [6]. Integrated care boards (ICBs) and NHS Trusts in England are required to work effectively with people and communities to ensure health and wellbeing for people, including in relation to the effects of inequalities, quality of health services for all individuals and the sustainable use of NHS resources [7]. Co‐production is an approach involving people and communities where there is a greater opportunity for people to have influence. The Coalition for Personalised Care [8] defines co‐production as ‘*a way of working that involves people who use health and care services, carers and communities in equal partnership; and which engages groups of people at the earliest stages of service design, development and evaluation*’. The voices of women should be heard in women's health service development and research, and there is vital importance in gaining inclusive perspectives [9, 10].

Although co‐production is increasingly advocated in health service design, much of the existing literature focuses on small‐scale projects, single services or conceptual frameworks, with limited empirical reporting on system‐level implementation within integrated care systems [11, 12]. There is a particular gap in evidence describing how co‐production can be operationalised across health systems while embedded within statutory NHS structures. This article outlines and presents the process undertaken to assess the local situation in one integrated care system Suffolk and North East Essex (SNEE). The authors would like to affirm that this article covers all people that need or use women's health services, including women, transgender men and non‐binary people registered as female at birth, and that women/woman will be used to describe the person in this article ([13]: ng23).

## 2 Background

In August 2022, the Women's Health Strategy for England set out the 10‐year ambitions for boosting the health and wellbeing of women and girls and for improving how the health and care system listens to women. Although challenging, the strategy encourages the expansion of women's health hubs across the country's 42 ICBs to review and improve access to service and health outcomes. Earlier in the same year, ICBs were given £595,000 non‐recurrent funding to establish the women's health hubs.

The task involved creating a hub by December 2024 providing support for menstrual problems, assessment and treatment; menopause assessment and treatment; contraception; pre‐contraceptive care; breast pain, assessment and care; pessary fitting and removal; cervical screening; and screening and treatment for sexually transmitted infections (STIs) and HIV.

In this ICB, SNEE allocated funds to include the co‐production and engagement, which amounted to £24,000, leaving a remaining budget of £571,000 for a service to be delivered (including rent, staff wages and consumables) while adopting a model that prioritised long‐term sustainability.

### 2.1 Women's Health Engagement

Patient and public involvement and engagement (PPIE) is very much integral and legislated [14] in health and social care research and should be included in areas of service improvement [15]. There is general agreement for more patient‐focused input with stakeholders using evidence to underpin improvements in services [9, 16]. Therefore, when there is a need to make improvements in a specific area and population, such as women's health services, it is important that representatives from those populations and those who use the local services are included in the co‐design and development of improvement of services that affect their lives [9, 17].

**FIGURE 1:**
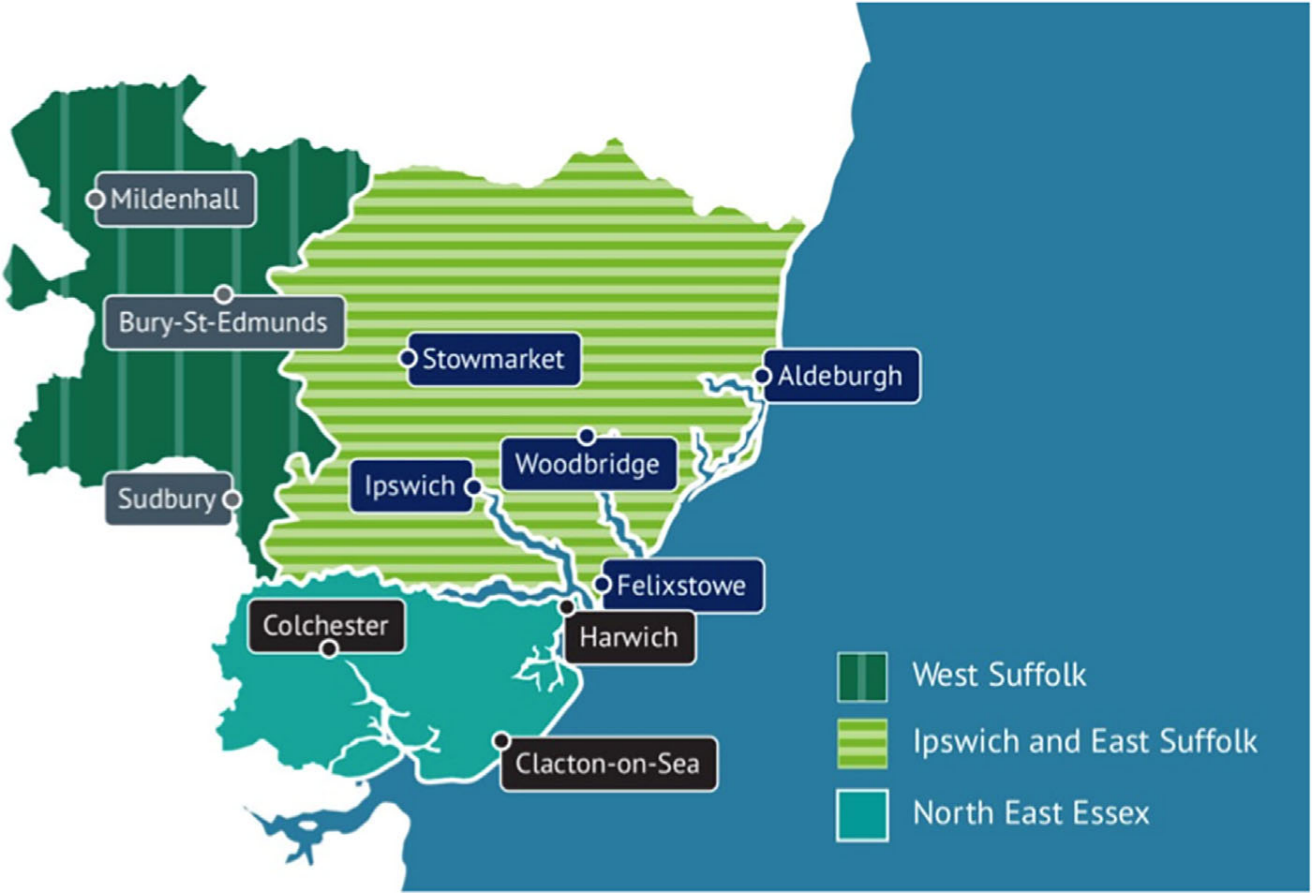
Suffolk and North East Essex (SNEE) geographical area.

Although national engagement had already taken place through the work of the strategy [1], SNEE wanted to ensure their plans as an integrated care system were right for their population. SNEE's geographical area covers a long coastline (see Figure 1) with several communities already identified nationally as experiencing inequalities [18].

SNEE ICB wanted to hear from as many people as possible, wanted to develop and maintain relationships and wanted to co‐produce the women's health programme with people and communities who contribute their lived experience to the programme. Therefore, the programme was thus built on the ‘*principle that those who use a service are best placed to help design it. It means aspiring to being equal partners and co‐creators*’ [19]. This principle was the underlying rationale which started the conversation in December 2023 between Healthwatch Suffolk and SNEE ICB, leading to the formation of a co‐production partnership and resulting programme.

This initiated a small advisory group which evolved to become the Women's Health Insight and Oversight Group (I&O Group) meeting monthly for two hours online until March 2025. This was an enthusiastic and innovative group that did more than advise, and the work continues to date through newly formed a Women's Health Network for SNEE with the original participants and a growing local network.

### 2.2 Theoretical Approach

The activities of the I&O Group were committed to being informed by a strong community approach of co‐production. Co‐production of public services, a concept developed in the 1970s [20], has been defined as, ‘*an umbrella concept*’ [21, p. 11], which moves culture and practice away from ‘doing to’ people towards ‘doing with’ [22]. Practical case studies and theoretical ideas only go so far in developing co‐production practice, the realities and relational dynamics should be experienced and practiced to be understood [23].

SNEE ICB utilised already‐developing practice, research and expertise in co‐production within the integrated care system. By learning from these and existing theory, such as the 45‐degree model for co‐production from the report ‘Transforming Society from Below’ [24] and integrated community care (ICC) [25], they built a model named the ‘45‐degree approach to co‐production’ [26]. This identifies that true societal transformation requires a combination of grassroots (horizontal) movements and institutional (vertical) support, empowering local communities to take control of issues that directly affect them through community development. It recognises that although bottom‐up initiatives are crucial, they are not sufficient on their own to address complex challenges without the contribution together from institutions [24, 27, 28], such as transformation and commissioning processes undertaken through ICBs.

ICC was developed by the Transnational Forum for ICC [25], and this refers to a ‘range of strategies to support professionals, organisations, policymakers and members of a community in a continuous process of co‐developing health, care and social support infrastructures and services’ [29, p. 106]. ICC aims to support frameworks and approaches of co‐production and co‐delivery to have equal contribution from the community. Together with the 45‐degree approach, this aims to shift decision‐making, service design, transformation and delivery closer to those communities that services are there to support. This shift is integral to the 10‐year health plan for England that expects local leaders to shape medium‐term plans that reflect the needs and ambitions of their communities and ensure that planning is informed by meaningful engagement with patients, carers, staff and local partners [30].

**FIGURE 2:**
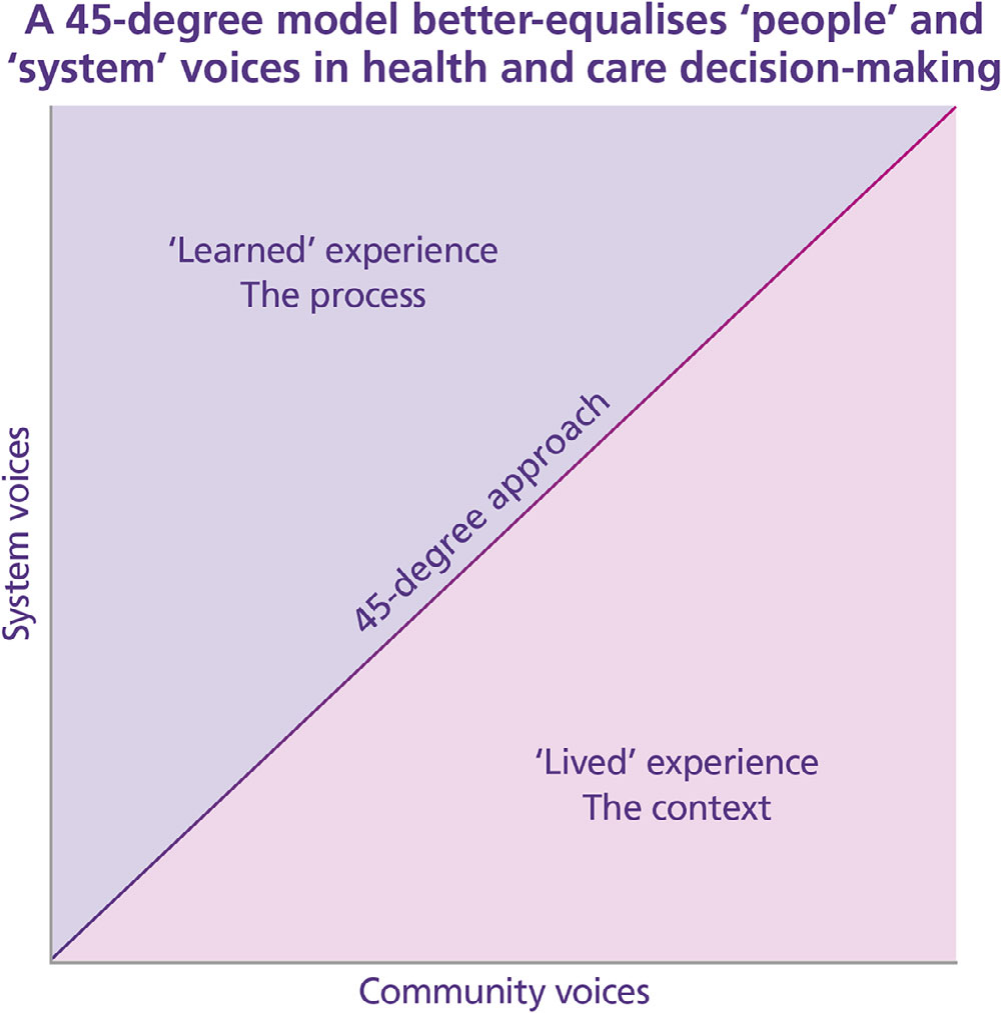
A depiction of the 45‐degree approach to co‐production.

A case study of co‐production using participatory action research applied Lawson's [24] 45‐degree model to integrated care and found that applying ‘*a balanced approach (in thinking, process and resourcing) between humans and systems […] between people's life experiences and skills, community‐centred approaches, and centralised system‐led approaches*’ [31, p. 312] would help ensure a more equal representation of lived and learned experience across service design and transformation (Figure 2). When working together, throughout the life cycle of a programme, it strives for equal representation and contribution from those with both ‘lived’ and ‘learned’ experience, who provide the relevant knowledge and context [31].

The women's health programme co‐production presented in this article is an example of these theoretical approaches applied in practice and has provided innovative organisational and developmental learning for this ICB and can inform other ICSs. These steps and methods are now presented in the following section.

## 3 Methods

### 3.1 Forming the Women's Health I&O Group

To develop the best possible services for our community in co‐production using the 45‐degree approach, SNEE ICB established the Women's Health I&O Group alongside a Steering Group. The I&O Group were recruited through existing networks of community and voluntary organisations, followed by word of mouth, to represent community voices and ‘lived experience’. The opportunity was advertised publicly, and anybody was welcome to join. The group gained over 160 members representing over 60 different organisations and community groups across SNEE, led in partnership by Healthwatch Suffolk and a team in SNEE ICB (L.M., L.N., S.C. and others). Key members representing the community were co‐production ambassadors of Healthwatch Suffolk who represented the transgender community and sickle cell experience. The Steering Group was established and comprised 24 members representing organisational leadership across the ICB, voluntary sector organisations, acute hospital trusts, local authorities, higher education institutions and Healthwatch to represent system voices and ‘learned experience’. The I&O Group and Steering Group had equal responsibility, both playing to their strengths and varying perspectives based on the 45‐degree model presented in Figure 2.

**FIGURE 3:**
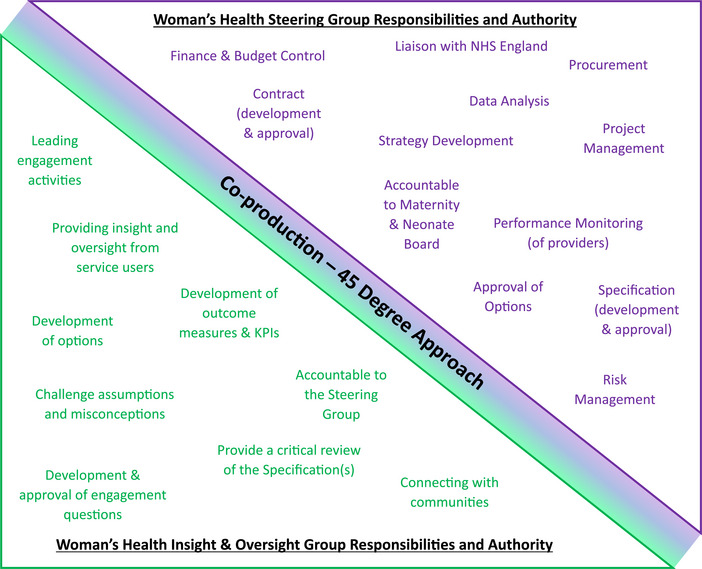
A presentation of the I&O Group and Steering Group responsibilities and authority in the women's health programme, as agreed and presented during the programme.

The Steering Group had an important leadership role in parallel to Lawson's [24] illustrative model, described by Sørensen et al. [28] as combining ‘hierarchical command’ with ‘webs of more informal interaction’ [28, p. 103]. This dynamic is presented in Figure 3, showing how elements of the women's health programme were mapped using the 45‐degree model, where the roles and responsibilities of the I&O Group and Steering Group were set out. This collaborative approach showed equal responsibility and authority across key elements of the programme by ‘community’ voices and ‘system’ voices, in accordance with the 45‐degree approach to co‐production. Both groups utilised their strengths and skills to ensure valuable contributions across the programme. For example, the I&O Group utilised trusted relationships with the communities they represented to engage to gain their insight to feed into the co‐production using accessible and inclusive methods appropriate to their audience, and the Steering Group developed the legal and financial contracts to procure the co‐produced services. One could not have developed the women's health programme without the other. The membership distribution, roles and responsibilities, and the facilitated approach explored above was designed to address some of the imbalance of power that often exists between ‘system voices’ and ‘community voices’, and further research and exploration of this would be beneficial.

As this programme was guided by national policy and funded by NHS England, the programme closely followed the national requirements and scope for co‐production. A National Women's Health Hub Core Specification [1] mandated the establishment of the hubs by December 2024. The hub (whether a physical site or virtual) could be designed around multiple different service offers and needs but must cover information, clinical support, consultations and triage services across all eight core service areas outlined in the national document (see Box 1).

**BOX 1 | National Women's Health Hub Core Specification [1]**

**Table:** 

1	Menstrual problems assessment and treatment (e.g., care for heavy, painful or irregular menstrual bleeding and care for conditions such as endometriosis and polycystic ovary syndrome)
2	Menopause assessment and treatment
3	Contraceptive counselling and provision of the full range of contraceptive methods, including long‐acting reversible contraception (LARC) fitting for both contraceptive and gynaecological purposes (for heavy menstrual bleeding and menopause); and LARC removal; and emergency hormonal contraception
4	Preconception care (e.g., supporting women to make informed choices about their health and well‐being prior to pregnancy)
5	Breast pain assessment and care
6	Pessary fitting and removal
7	Cervical screening
8	Screening and treatment for sexually transmitted infections (STIs) and HIV screening

The I&O Group met monthly to progress the co‐production in line with the national direction, ensuring community participation.

### 3.2 The Service Improvement

**FIGURE 4:**
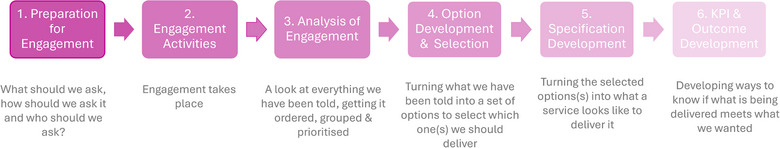
Phases of co‐production.

As this programme followed a service improvement design, it did not require ethical permission but received approval from the local ICB. The co‐production process was guided by evidence‐based theory and a six‐phased approach outlined in Figure 4. Each of the six steps is discussed below.

The I&O Group started by deciding together that all six phases of the programme should be co‐produced (Figure 4). This started with the I&O Group co‐producing a set of targeted engagement questions (1) for different demographics within our community to help us start our local service development process. This was to ensure whatever services we designed to implement the strategy reflect what is important and valuable to people. The group engaged with their communities using the questions to gain their insights to shape the services (2). Together the I&O Group discussed the findings of the engagement work and started to co‐produce and define what options the programme could offer and develop (3). Twelve service options were then developed, presented and discussed. The I&O group were given time to process this and to discuss further, and then options were voted on and the group decided to take five options forward (4). Each of the five options had allocated groups who worked through the specific tasks, feeding through discussion and members of the groups co‐produced service specifications (5). The SNEE ICB team led on the setup and procurement of services. As the projects started to go live, the I&O Group collaborated and co‐produced how to measure and evaluate each of the five projects (6). Some of these were with members through evaluative workshops with the local population. All five projects were live by January 2025.

### 3.3 Engagement Tools

By co‐producing the questions for the engagement as presented in Box 2, it ensured their accessibility and understandability and allowed for peer‐to‐peer testing of the questions before launch. The survey was distributed within the community, and responses were kept anonymous for those taking part to speak freely about their experiences, without fear or judgement. The survey was launched online on Friday 1 March 2024 and closed on Friday 19 April 2024. Participants read the accompanying information and, by submitting the survey, agreed their anonymous answers could be used within the data analysis. Community leaders and organisation representatives shared the information and provided opportunities to complete the survey to ensure a bespoke and accessible approach as required. There were a total of 1238 responses to the engagement questions in total. Authors S.C. and L.M., representing Healthwatch and the NHS, thematically analysed the responses and coded them into difficulties and barriers, positive experiences and ideas for women's healthcare to be better, as per the questions.

**BOX 2 | The co‐produced engagement questions**

**Table:** 

1	What difficulties and barriers have you experienced throughout your life when trying to locate or access women's healthcare?
2	What positive experiences have you had throughout your life when accessing women's healthcare?
3	What ideas do you have for women's healthcare to be better? What needs to change?
4	How did you hear about this survey? (If you know, please include which organisation asked you to complete it)
5	Any of the following ages? Please take which, and add any further detail or thoughts in the comment box 10–15 16–24 25–45 46–65 66+ Not specific to any age Other, please specify
6	Are you any of the following gender identities? Please take which, and any further detail or thoughts in the comment box Female Male Transgender female Transgender male Gender variant/non‐conforming Not specific to any gender Other (please specify)
7	Do you speak or use languages other than English? If yes, please add details
8	Are you from any specific ethnicities, cultures or have religious beliefs? If yes, please add details
9	Do you have any special physical disabilities, learning disabilities or sensory impairment
10	Are you a family carer? Do you provide unpaid care to a family to my friend or to my family member (you do not have to be living with them)? If yes, please add details

## 4 Findings

The key findings from the three questions as part of the engagement survey are presented below, and descriptive analysis is used to present the results.

### 4.1 Question One: What Difficulties and Barriers Have You Experienced Throughout Your Life When Trying to Locate or Access Women's Healthcare?

**FIGURE 5:**
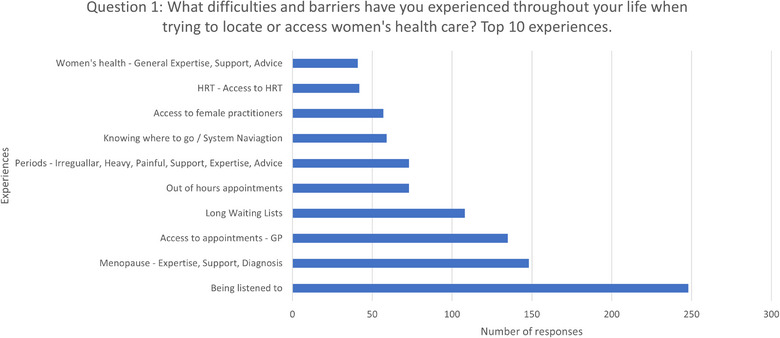
Top 10 difficulties and barriers in accessing women's healthcare.

A total of 1174 people (95%) answered this question, as presented in Figure 5. Among those who responded, 1908 difficulties or barriers were mentioned (with some individuals mentioning more than one) and were categorised into 125 themes. Of these, 85 themes had 10 or fewer mentions of the difficulty or barrier, which is less than 1% of the total respondents.

The most mentioned issue, ‘*Being Listened to*’, was brought up 245 times, which is almost 20% of all respondents. However, ‘*Being Listened to*’ was also cited as a positive experience by 90 respondents (7%), meaning that despite their difficulty or barrier, those individuals did feel listened to.

Notably, ‘*Being Listened to*’ is the fourth most popular suggestion for improving women's healthcare.

### 4.2 Question Two: What Positive Experiences Have You Had Throughout Your Life? When Accessing Women's Healthcare?

**FIGURE 6:**
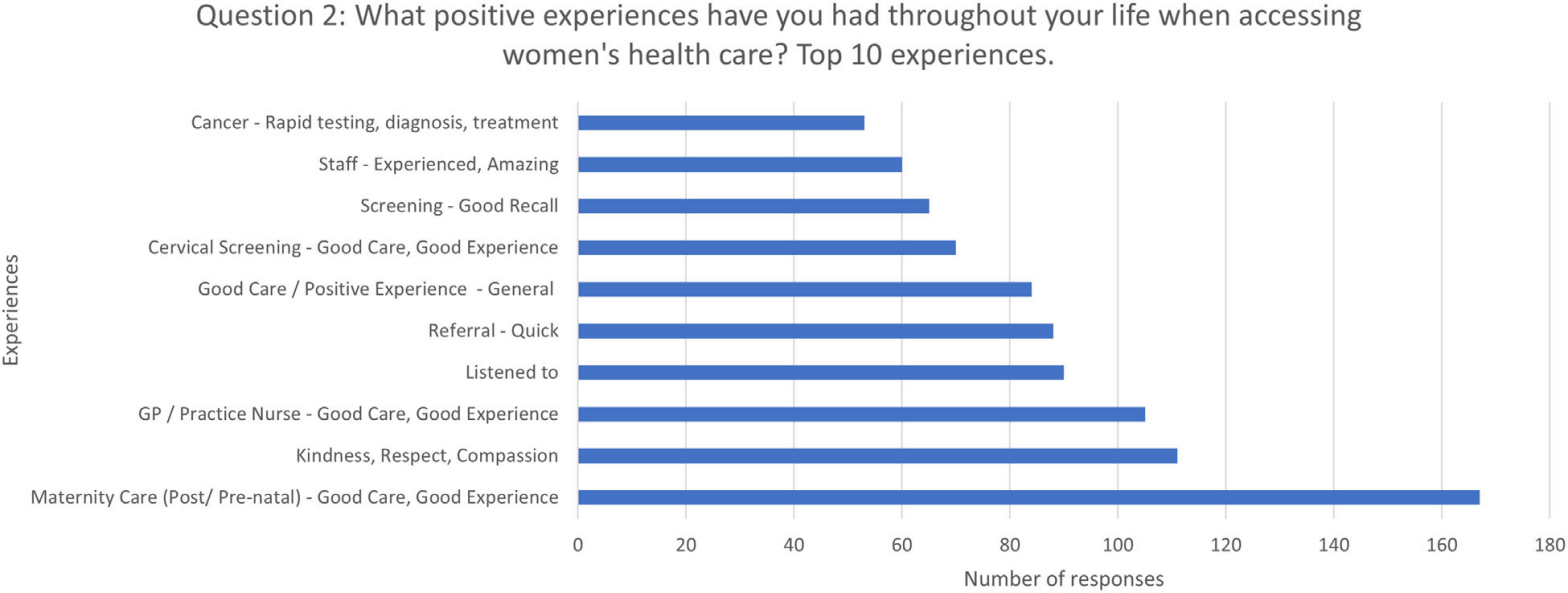
Top 10 positive experiences in accessing women's healthcare.

The question was answered by 1102 people (see Figure 6), which accounts for 89% of the total respondents. Among these answers, there were 1578 positive experiences mentioned, with some people mentioning more than one. These experiences were categorised into 79 themes. Of these 49 themes, 10 or fewer mentions had positive experiences, which is less than 1% of respondents.

The most popular response, ‘*Maternity Care (Post/Pre‐natal)—Good Care, Good Experience*’, was mentioned 167 times, accounting for 13% of respondents.

### 4.3 Question Three: What Ideas Do You Have for Women's Healthcare to Be Better? What Needs to Change?

**FIGURE 7:**
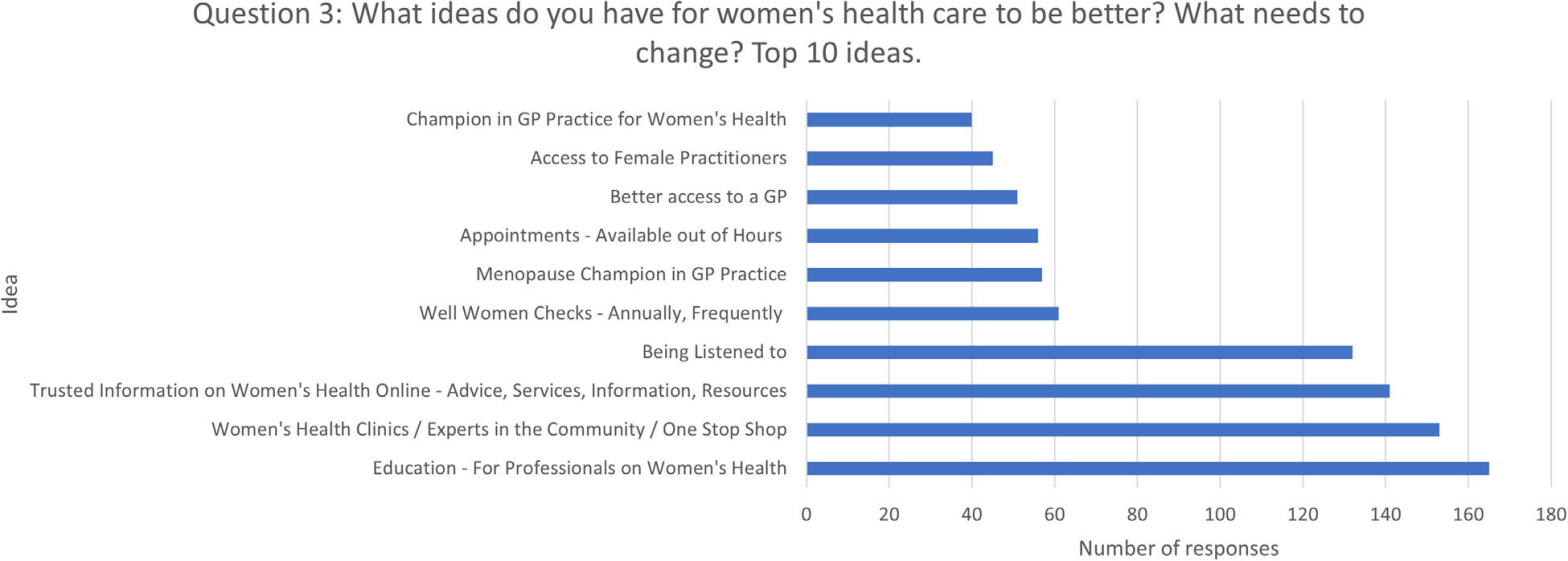
Top 10 ideas for improving access to women's healthcare.

Out of 1142 people surveyed, 92% answered the question (see Figure 7). Among the responses, there were 1822 different ideas and suggestions for what needs to change (some people mentioned more than one idea). These were categorised into 133 themes. Of these 94 themes, 10 or fewer mentions (which is less than 1% of the respondents), the most popular response, ‘*Education—For Professionals on Women's Health*’, was mentioned 165 times (13% of respondents). This category included education on menopause, periods, endometriosis, PCOS, fibroids and general education on women's health.

The survey outcomes informed and enabled the development of 12 localised service options, based on realistic procurable service provision decided by the Steering Group and NHS commissioners, thus responding to population needs, and these are presented in Box 3.

**BOX 3 | The 12 localised service options**

**Table:** 

1	Option A: An Information Website and App
2	Option B: A Signposting and Self‐Help Website and Support Line
3	Option C: GP Practice Professional Education on Women's Health
4	Option D: GP Practice Menopause Champion
5	Option E: Menopause Group Clinic
6	Option F: Fixed Location One Stop Shop x3
7	Option G: Pop‐up One Stop Shop/Clinic
8	Option H: Long‐Acting Reversible Contraception (LARC) for Non‐Contraceptive Purposes
9	Option I: Training for LARC Fitters
10	Option J: Expansion of the VIP Service
11	Option K: Gynae Treat and Triage Service
12	Option L: Research in Women's Health

The outcomes of the survey were shared with the Women's Health I&O Group along with the options for services. The I&O Group were asked to read the detailed options and be prepared to vote for their preferred options. The groups were reminded that they could keep more than one option, but they were advised that not all options presented were affordable. A set of principles was also developed by the I&O Group and agreed upon. These principles were featured in all the options and must be trauma‐informed, accessible (easy read, access to translators/interpreters, ramps, etc.), striving to always have a listening culture, culturally sensitive and transgender and non‐binary inclusive. These principles must also aim to reduce health inequalities, ensure people have the best level of care and a good experience and improve outcomes for our population.

A total of 86 members of the I&O Group took part in a vote for their preferred options for the offer, as per the number of people attending the relevant I&O Group meeting or taking part via email. These votes came from a good cross‐section of our I&O Group membership of representative members from the ICB, Voluntary, Community, Faith and Social Enterprise (VCFSE), smaller community groups and people with lived experience, hospitals, primary care, sexual health and wider community services. Five most popular options as a result of the voting are as follows, with the most popular option voted for is *to develop and offer GP Practice Professional Education on Women's Health (Option C)*. In second place was *the development of an Information Website and/ or App that provides self‐help, information, and advice videos (Option A)*. In third and fourth place are the *Menopause Group Clinic (Option E)* and *the Menopause GP Practice Champion (Option D)*. In fifth place is the development of *a Signposting and Self‐Help Website and Support Line (Option B)*. Finally, in sixth place is the *Pop‐up One Stop Shop/Clinic (option G)*, which was far more popular than the same offer in a fixed location (Option F) (which came in 10th place).

The I&O Group was aware that all 12 service options were not financially viable, so through discussion, feedback and evaluation, while considering the project principles, the following five services were developed locally.

1. A women's health app.
2. Virtual menopause group clinics.
3. GP practice menopause champion training.
4. Community provision of vaginal ring pessaries in the community and long‐acting reversible contraception (LARC) for non‐contraceptive purposes (such as heavy menstrual bleeding).
5. GP education and training on women's health.

### 4.4 Evaluation of Co‐Production

As part of this work an informal mixed methods evaluation survey was designed with the co‐facilitators of the I&O Group and distributed to the groups. Despite the long‐term involvement of members of the group and several reminders and follow‐ups, this part of the evaluation of the co‐production received only nine responses. However, the responses covered a range of members from the I&O and Steering groups and demonstrated varying levels of experience of the co‐production process.

The evaluation showed that for those that responded, the co‐production experience was overall positive, with reported feelings of the process being inclusive and engaging for a variety of stakeholders. Respondents also critiqued the limitations and suggested improvements for the co‐production, such as the limitations of a large group size, reliance on the facilitators to progress a large amount of work between meetings, and time and commitment required from community groups. The survey also collected some qualitative comments, which are themed and presented in Table 1.

**TABLE 1:** Themed qualitative responses from the co‐production evaluation.

Question	Themes	Quotes
Can you think of any key benefits of the I&O Group model for co‐production?	*Connection*	*‘A good way to connect with a vast array of stakeholders and interested individuals’* *‘A good way to involve as many groups and individuals as possible in the development of large scale programmes’* *‘Kept everyone connected and a feeling of equality without burdening one particular group’* *‘Very positive way and delivering outputs for the local area’*
	*Experience*	*‘Experience and knowledge from a range of sectors’* *‘Based on theory and open to learn’* *‘People inputted for the first time into women's health services—a sustainable model’* *‘I think that the work done has been managed and run very efficiently in the time allowing. I have been so impressed’*
	*Inclusive*	*‘Very inclusive’* *‘Opinions and input from really wide range of communities/clinicians/voluntary organisations’*
Can you think of any limitations of the I&O Group model for co‐production?	*Size of group*	*‘The size of the membership and online format is daunting and inaccessible for some’* *‘A lot of work coming from the members for a project lead to take on and consider’* *‘The scale of the group is vast, and the many areas of need is overwhelming’* *‘With bigger groups it takes a longer time to reach a decision’*
	*Time and pace*	*‘Some members of the VCSFE sector don't have the time or resource to be as fully involved as in this work as they might like’* *‘It relies a lot on I&O Group members to volunteer time to join, contribute and engage with those they support. Some won't manage this’* *‘Pace was fast, and time/commitment was big. Not all could work within this model in the absence of reward’*
Can you think of any ways that the I&O Group could have been improved for you (or the community you represent)?	*Tight timescale*	*‘The pace and detail was quite fast and high level for some so they would have dropped off’* *‘Timescales were quite tight to allow fully for this work’* *‘It was on a tight timeline; communication needs to be open and getting that in our in social media and repeating it’* *‘I feel that perhaps slightly too much time was given to the initial planning and surveys which went out and less in planning the options/ implementation’*
	*More support*	*‘It has been a great start, but it would be good to resource and allow for some members to REALLY involve their communities in this work and the task and finish groups’* *‘More support and resource to VCFSE [Voluntary, Community, Faith, and Social Enterprise] groups to involve people in communities to develop programmes and help make even more accessible and fit for purpose’*
Can you think of any ways that working alongside the I&O Group differs to other models of working for you?	*New and collaborative*	*‘A new experience and very valuable, very positive and happy to use this model in future projects’* *‘I tend to not get the chance to work collaboratively so this is brilliant’*

The survey also asked about the respondents’ experience of being part of the women's health programme, with the majority stating that this was ‘*a valuable use of my time*’, that ‘*it built knowledge and skills in co‐production*’ together with ‘*the process and resulting outcomes*’. There seemed to be some variability in how ‘*respondents felt their views were represented*’ *and how* “*their community was represented*”. Respondents appeared ‘*confident with the I&O Group*’, “*were motivated to attend*” and ‘*were co‐facilitated well between organisations*’.

Due to the small response size of the evaluation, it is challenging to draw conclusions, and facilitators have learnt that more discussion‐based methods of evaluation may have been more suited to this group to match their meeting‐based involvement within the programme itself.

In summary, the engagement process yielded 1238 responses to a co‐produced survey, generating rich data on women's experiences and priorities across SNEE. Analysis revealed 1908 instances of difficulty in accessing services, with the most cited barrier being not feeling ‘*listened to*’, followed by access delays, lack of continuity in care and limited specialist knowledge in primary care. Positive experiences (*n* = 1578) were also recorded, with high satisfaction reported in maternity and postnatal services, highlighting the importance of compassionate care and relational continuity. Respondents proposed 1822 suggestions for improving services, with the strongest theme being *the need for education and training for healthcare professionals* on women's health issues such as menopause, endometriosis and menstrual disorders.

These insights directly informed the development of 12 service options, later prioritised by vote from members of the I&O Group. The top six included GP education, a menopause group clinic, a signposting website and mobile clinics. Final services implemented included training for GP Menopause Champions, provision of LARC for non‐contraceptive use, and a digital self‐help platform. These priorities reflect a community‐driven approach that centres access, knowledge and equity in service design, highlighting the value of participatory models in responding to complex, unmet health needs.

## 5 Discussion

### 5.1 Responding to a National Initiative

This programme demonstrates how a local ICS can respond effectively to national policy by embedding participatory models of service co‐production and reflects on the response to the national initiative to redesign women's health services using inclusive and person‐centred approaches. The work around Women's Health Hubs emphasised improving access, listening to women's voices and addressing historically embedded inequalities in health outcomes. In this programme, we have used a structured, evidence‐based approach to engagement drawing on a six‐phase co‐production process using the 45‐degree model to enable meaningful contributions from both system stakeholders and individuals with lived experience [31]. The large‐scale community survey (*n* = 1238) provided localised insight into gaps in care, and these findings directly shaped service delivery models. Critically, this work answers recent calls in the literature for amplifying women's voices in the planning and evaluation of services related to menopause, reproductive care and health literacy [9].

### 5.2 Scale and Diversity

A defining strength of the programme was the scale and diversity of engagement achieved through the I&O Group. With over 160 members across 60+ organisations, the group encompassed a wide range of community perspectives, many of whom had not previously been engaged in health planning processes. This structure helped operationalise the 45‐degree model, ensuring a balance between community knowledge (horizontal insight) and institutional power (vertical systems) [24, 31]. The decision‐making process was shared between the I&O Group and Steering Group, and together, these bodies co‐designed interventions that were responsive to local health needs, such as community‐based LARC provision, menopause group clinics and culturally inclusive digital resources. Participants also co‐produced the engagement questions, voted on service priorities, disseminated information and helped shape evaluation tools, demonstrating a model of co‐production that was both strategic and grassroots in orientation. To retain momentum and ability to co‐produce programmes in the future, the I&O Group membership continues to grow through a newly formed Women's Health Network [26].

### 5.3 Programme Challenges and Limitations

The article presents a strong, theoretically grounded example of co‐production in women's health service design, but several challenges and key limitations emerge on closer analysis. These can be grouped into methodological, practical and strategic categories.

One significant challenge encountered was the time and pace of the project, which was developed over a tight timeline with a non‐recurrent budget, posing risks for sustainability of the resulting services. Although there is no clear strategy presented in this article for securing future investment or embedding services into mainstream provision, impact has been made in this ICB, and an application and a case for recurrent service provision have been made on the basis of this evidence.

Although the online format enabled wide participation, this article itself acknowledges that the digital format was inaccessible or overwhelming for some, especially smaller voluntary, community, faith and social enterprise (VCFSE) organisations. Some groups lacked the time, capacity or resources to fully engage, which introduces possible participation bias, with those most able to participate shaping decisions, potentially under‐representing marginalised voices. However, services involved played an important role in reaching out to those who may not have been represented. These issues align with known structural barriers to equitable engagement in participatory health work [32]. Health inequalities in coastal communities such as SNEE have been well documented, with higher rates of preventable illness and reduced access to healthcare services [18]. Ensuring future co‐production efforts address these inequalities requires dedicated, ongoing funding and infrastructure to support the involvement of under‐represented populations not just during the design phase, but throughout implementation and evaluation.

Despite strong engagement during early phases, the actual decision‐making and prioritising of service options were based on 86 votes from a subset of the full group. The programme was of a long duration, and although attendance varied, communication via email was maintained with all participants. The process, while inclusive in design, may not have reached deep engagement with all intersectional identities (e.g., disabled women and LGBTQ+ individuals); this was acknowledged by the I&O Group and revisited on many occasions in the process, especially given the fast pace and voluntary nature of involvement. Further mapping could have been initiated with group members to record which demographics and experiences were both represented in the group and engaged with. Although there is limited depth in the evaluation of the co‐production process with just nine survey responses, despite a very large participant pool (over 160 members), there were detailed responses in the qualitative feedback on the process and co‐production experience.

Although this article does not compare with other service improvement models or co‐production approaches, the aim was to present, disseminate and share these good practices. Although it may be difficult to understand how different processes might yield different outcomes, and there is no pre‐programmed data presented and therefore limited evaluative claims, time was spent understanding the local population data and scoping the existing local women's service provision.

Nonetheless, the programme offers a promising model for health systems aiming to transform services through co‐production. It demonstrates how the integration of lived experience and professional expertise through respectful, well‐structured partnerships can result in health service redesign that is more inclusive, evidence‐informed and locally appropriate. Although limitations remain in terms of reach and sustainability, this work affirms the importance of embedding co‐production as a core mechanism for addressing both service fragmentation and health inequality. The co‐design of five new women's health services across the ICS exemplifies what is possible when communities are not only consulted but are also co‐authors in shaping solutions. As national strategies continue to evolve, local systems must build on this momentum, ensuring that co‐production is not a one‐off initiative but a fundamental design principle in health equity.

## 6 Conclusion

The Women's Health I&O Group demonstrates how co‐production can be operationalised at Integrated Care System level to inform women's health service improvement. By integrating lived experience with professional expertise, the programme identified local priorities and supported the development of five women's health service models aimed at improving access and equity.

The initiative faced challenges, including non‐recurrent funding, time constraints and potential under‐representation of some population groups. These limitations highlight the need for sustained engagement, broader inclusion strategies and longer‐term evaluation.

Future work will focus on evaluating service outcomes, assessing the impact on access and experience and exploring the transferability of this approach to other settings. Further research is needed to understand how system‐level co‐production can be sustained over time and embedded within routine service planning across integrated care systems.

## Author Contributions

**Susan Conquer**: conceptualisation, methodology, project administration, writing – original draft, writing – review and editing. **Lizzie Mapplebeck**: data curation, methodology, project administration, resources, writing – review and editing. **Annetta Bradshaw**: supervision. **Lisa Nobes**: conceptualisation, funding acquisition, resources, supervision. **Camille Cronin**: supervision, writing – original draft, writing – review and editing.

## Funding

The work was supported by Department of Health and Social Care.

## Consent

Participation was voluntary, and completion of the survey or engagement activities was taken as implied consent for the use of anonymised data.

## Conflicts of Interest

The authors declare no conflicts of interest.

## Data Availability

The authors have nothing to report.
